# Lichtenstein technique for inguinal hernia repair: ten recommendations to optimize surgical outcomes

**DOI:** 10.1007/s10029-024-03094-w

**Published:** 2024-06-20

**Authors:** Bruno Amantini Messias, Rafael Gonçalves Nicastro, Erica Rossi Mocchetti, Jaques Waisberg, Sergio Roll, Marcelo Augusto Fontenelle Ribeiro Junior

**Affiliations:** 1 Department of Surgery, General Hospital of Carapicuiba, 95 Pedreira Street, Carapicuiba, SP 06321-665 Brazil; 2 Department of Surgery, São Camilo University Center, São Paulo, SP Brazil; 3 Department of Surgery, State Public Servant Hospital (IAMSPE), São Paulo, SP Brazil; 4 Department of Surgery, ABC Medical School, Santo Andre, SP Brazil; 5 Abdominal Wall Surgery Unit, Santa Casa de São Paulo, São Paulo, SP Brazil; 6 Hernia Center, Oswaldo Cruz German Hospital, Sao Paulo, SP Brazil; 7 Division Chair Trauma, Critical Care and Acute Care Surgery, Sheikh Shakhbout Medical City, Mayo Clinic, Abu Dhabi, United Arab Emirates; 8 Department of Surgery, Catholic University of São Paulo, Sorocaba, SP Brazil

**Keywords:** Inguinal, Hernia, Lichtenstein, Surgery

## Abstract

**Purpose:**

Approximately 20 million individuals worldwide undergo inguinal hernia surgery annually. The Lichtenstein technique is the most commonly used surgical procedure in this setting. The objective of this study was to revisit this technique and present ten recommendations based on the best practices.

**Methods:**

PubMed and Scientific Electronic Library Online were used to systematically search for articles about the Lichtenstein technique and its modifications. Literature regarding this technique and surgical strategies to prevent chronic pain were the basis for formulating ten recommendations for best practices during Lichtenstein surgery.

**Results:**

Ten recommendations were proposed based on best practices in the Lichtenstein technique: neuroanatomical assessment, chronic pain prevention, pragmatic neurectomy, spermatic cord structure management, femoral canal assessment, hernia sac management, mesh characteristics, fixation, recurrence prevention, and surgical convalescence.

**Conclusion:**

The ten recommendations are practical ways to achieve a safe and successful procedure. We fell that following these recommendations can improve surgical outcomes using the Lichtenstein technique.

## Background

Inguinal hernias (IH) constitute 75% of abdominal wall defects, with a lifetime risk ranging from 27 to 43% in men and 3–6% in women [1, 2]. IH surgery is one of the most common procedures worldwide, with an estimated 20 million individuals undergoing it annually [1].

The Lichtenstein tension-free technique was introduced in 1984 by Dr. Irving Lichtenstein, who aimed to eliminate the adverse effects of suture tension observed using previous techniques. Understanding the metabolic origin of IH (i.e., collagen metabolism dysfunction and type 1/type 3 collagen ratio) is pivotal in developing this technique [2–9]. The Lichtenstein technique involves placing a polypropylene mesh between the floor of the inguinal region and the aponeurosis of the external oblique muscle (EOM). This mesh eliminates the need for tension sutures and the use of compromised tissues to repair IH. Increased intra-abdominal pressure during effort leads to EOM contraction, which exerts counterpressure on the mesh, thus effectively utilizing the intra-abdominal pressure for repair [3, 6]. The surgical outcomes of this technique have proved highly promising, with a recurrence rate of less than 1% [5, 6, 8, 9].

A review of the initial cases identified four cases of recurrence (three resulting from juxtaposed mesh fixation in the pubic symphysis and one from ruptured mesh fixation in the inguinal ligament). Given these technical flaws, Amid et al. (1989) proposed modifications, including increasing the mesh size (7.5 × 15 cm), a 2 cm overlap in the pubic tubercle region, crossing the mesh edges in the spermatic cord (SC), and using interrupted stitches on the upper edge of the mesh. The proposed modifications enhanced surgical outcomes and resulted in the current Amid-modified Lichtenstein technique, a globally recognized surgical procedure [5–8, 10–16].

This technique presents five principles based on the dynamic physical characteristics of the abdominal wall and intra-abdominal pressure. The principles are influenced by modified intra-abdominal pressure, which can vary from 8 cm of water [H_2_O] when supine to 80 cm H_2_O with physical effort and mesh shrinkage in living tissue, resulting in contraction. Most authors describe the mesh shrinkage as approximately 20%. Shrinkage is linked to the scarring of the recipient tissue, which leads to mesh contraction as the tissue heals [4, 6, 13].

The five principles described by Lichtenstein are as follows: (i) use of a footprint-shaped mesh measuring approximately 7.5 × 15 cm, with a medial overlap of 2 cm in the pubic symphysis region, 3–4 cm above the inguinal triangle and 5–6 cm lateral to the internal inguinal ring; (ii) crossing the mesh edges behind the SC to avoid lateral recurrence; (iii) suturing the mesh with two separate stitches to the sheath of the rectus abdominis muscle and to the aponeurosis of the internal oblique muscle (IOM) to prevent iliohypogastric (IHG) nerve injury and suturing the lower edge of the mesh to the inguinal ligament with continuous nonabsorbable suture (passing the needle three to four times) to prevent mesh mobilization; (iv) maintaining the mesh slightly relaxed or shaped like a dome to contain transversalis fascia protrusion during physical effort, thus compensating for mesh counter-traction; and (v) visualizing and protecting the three inguinal nerves: the ilioinguinal (II) nerve, the IHG nerve, and the genital branch of the genitofemoral (GNF) nerve.

Recent data from Brazil reveals that almost all (99.2%) of the more than 700,000 IH surgeries conducted with the Unified Health System between 2017 and 2022 employed the open technique [15]. A recent population study with over 260,000 patients in Spain indicated an open surgery rate of 94.3% [17]. The review led by the *Americas Hernia Society Quality Collaborative* also highlighted a significant rate of open surgery (42%) among North American surgeons [18]. Although precise recent data from all countries are unavailable, the Lichtenstein surgery remains the preferred choice for most surgeons [16]. The American College of Surgeons endorsed the Lichtenstein technique as the gold standard surgery [3, 6] for IH, and the main consensus statements from hernia and abdominal wall societies currently recommend the Lichtenstein technique as the preferred surgical approach for anterior IH repair with mesh [1–3, 5, 6, 10–12, 19].

Literature regarding technical steps of the Lichtenstein surgery, modifications, and chronic pain prevention measures are the utmost importance to achieve a safe and successful procedure. Studies have shown the need to improve neuroanatomical knowledge of the inguinal region and the technical steps of the Lichtenstein technique [12–16, 20]. This study aims to present ten recommendations grounded in the five principles of the Lichtenstein technique, supported by current scientific evidence. These recommendations aim to revisit the technique and outline best practices for treating IH using the Lichtenstein technique.

## Methods

This descriptive-analytical study was approved by the Research Ethics Committee of our university. PubMed and Scientific Electronic Library Online (SciELO) were used to systematically search for articles about the Lichtenstein technique and its modifications. The search included studies published in English or Portuguese between 1987 and 2023. Literature regarding this technique and surgical strategies to prevent chronic pain were the basis for formulating ten recommendations for best practices during Lichtenstein surgery.

## Results

This study presents ten recommendations for treating IH using the Lichtenstein technique. Following we described detailed 10 recommendations we consider a practical guide so that surgeons with all levels of expertise can achieve a safe and successful procedure.

### Neuroanatomical assessment

#### Recommendation 1

Identification of the II nerve, the IHG nerve, and the genital branch of the GNF nerve.

**Fig. 1:**
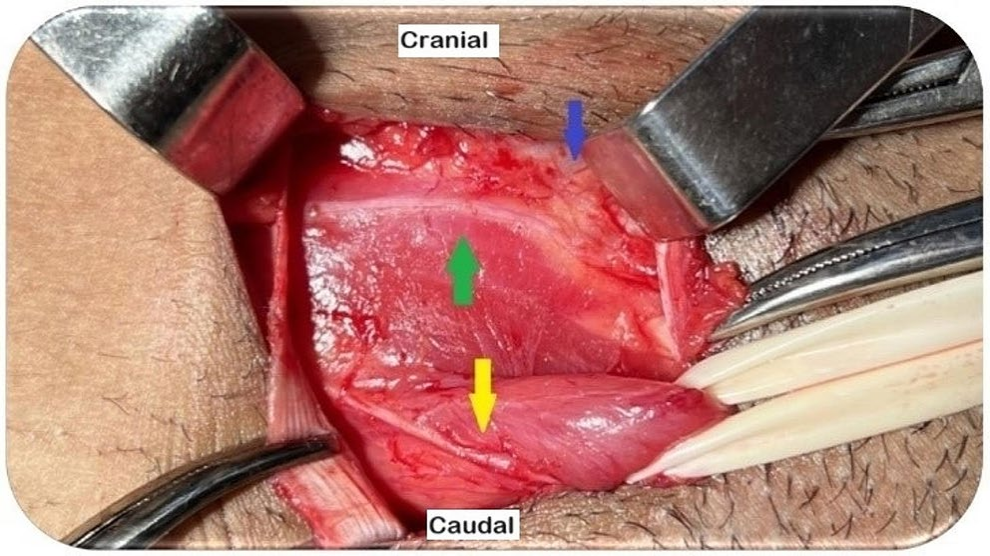
Right inguinal region post-EOM aponeurosis opening. Identification of the II and IHG nerves. (Source: author). Green arrow: IHG; yellow arrow: II; blue arrow: IOM aponeurosis

**Fig. 2:**
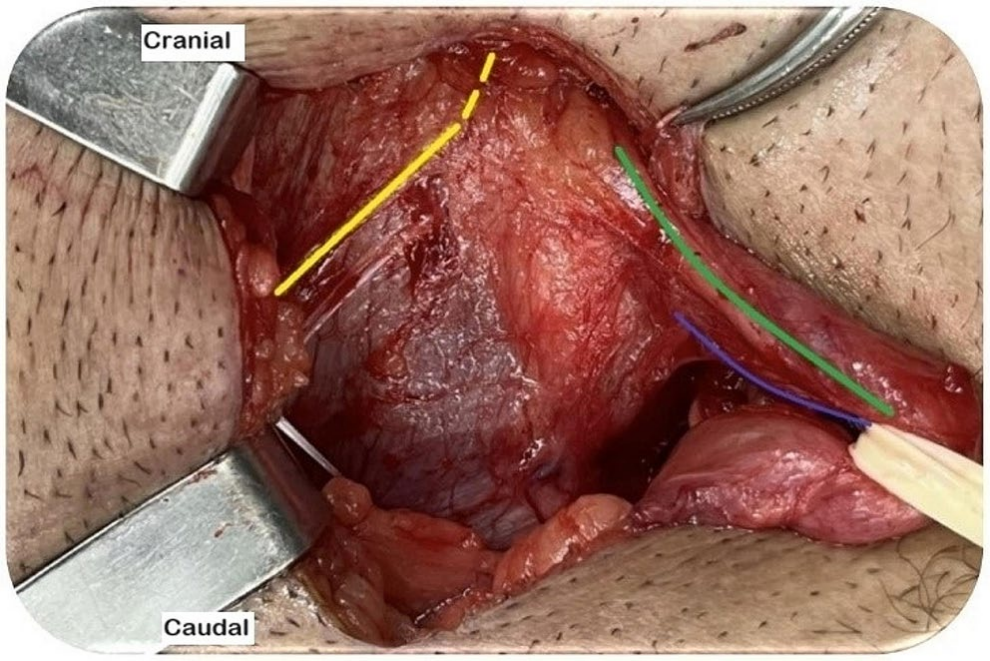
Left inguinal region post-EOM aponeurosis opening. The three nerves schematically identified. (Source: author). Yellow line: the IHG nerve. Green line: the II nerve. Blue line: the genital branch of the GNF nerve

Identifying the three nerves is fundamental step in IH surgery. (Figures 1 and 2) [1, 4–6, 8, 12–14]. These nerves can be identified by open repair in 70–90% of cases [20, 21, 23]. Although this technical step can increase the surgical time by approximately five minutes, it offers countless benefits to patients with chronic pain [21–23]. 

The II nerve is the most anterior and is easily identifiable at the opening of the EOM aponeurosis. It penetrates the IOM into the inguinal canal, runs ventrally parallel to the SC, and exits through an external inguinal ring. The IHG nerve is identified when the EOM layer is separated from the IOM. It has a visible part that passes laterally through the IOM aponeurosis (approximately 2.4 cm) and a hidden part that passes through the IOM fibers [20, 21, 23]. The genital branch of the GNF nerve, which has a small diameter, is the most challenging nerve to identify. In men, it generally penetrates the deep inguinal ring and descends to the lateral caudal zone of the SC, whereas in women, it follows the round ligament. The external cremasteric vein (blue line sign) is an anatomical landmark that helps identify the genital branch as they run alongside each other [16, 20, 21, 23, 24]. 

Several anatomical variations should be highlighted, including the early superficialization of the II nerve in the EOM aponeurosis between the deep and superficial rings, fusion of the II and IHG nerves or absence of one of these nerves, the aberrant II branch descending alongside the genital branch of the GNF, and the II nerve within the cremaster muscle, which is identifiable in up to 35% of patients [20, 23, 24].

### Chronic pain prevention

#### Recommendation 2

Comprehensive and meticulous dissection, proper identification, and dissection of nerves, and covering fascia protection.

**Fig. 3:**
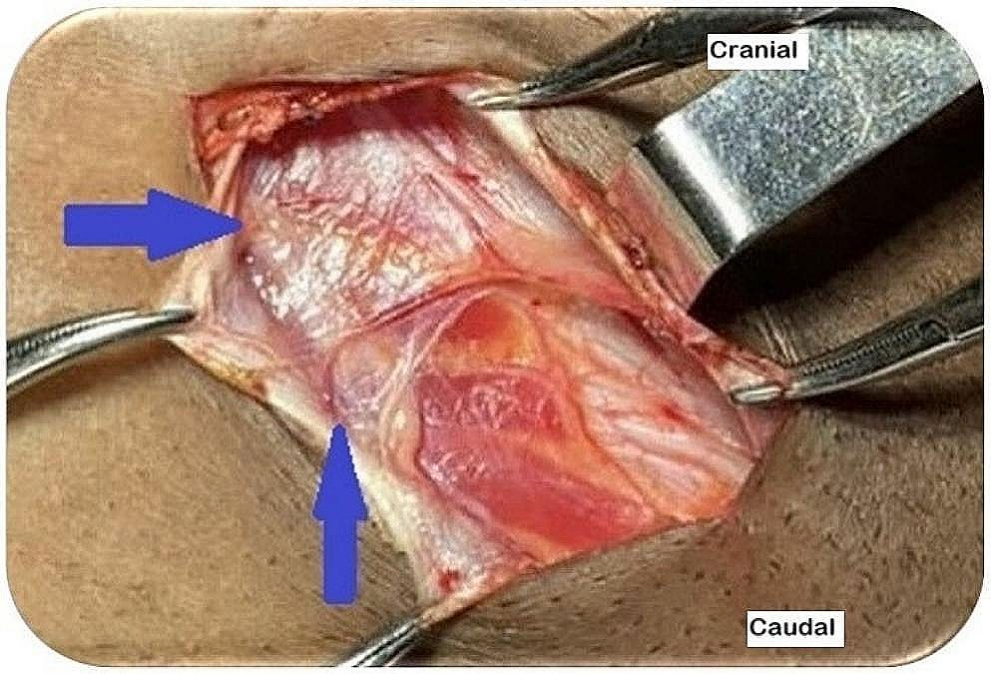
Right inguinal region post-EOM aponeurosis opening illustrating identification and preservation of the nerve protection fascia (blue arrow). (source: author)

The surgical procedure begins with meticulous dissection of the inguinal region because complications such as hematoma, seroma, and infection are risk factors for chronic pain. Comprehensive dissection of the inguinal region is crucial. Minimal dissection, which may reduce the duration of the surgical procedure but compromise nerve identification, should be avoided [16, 20]. The subcutaneous cellular tissue is carefully dissected to avoid injuring the II and IHG nerve branches, which may be prematurely found in this topography. Careful opening of the EOM aponeurosis prevents inadvertent injury to the II nerve. The II and IHG nerves should be kept in their usual beds without manipulation to avoid damaging the neurolemma. The protective fascia (connective areolar tissue present in the IOM) covering these nerves should be kept intact (Fig. 3). This is achieved by dissecting the fascia from the upper and lower edges of the EOM aponeurosis as close to the aponeurosis as possible. This connective tissue prevents direct contact between the mesh and the nerve [16, 20, 23, 24].

Preserving the deep cremasteric fascia during SC dissection is vital because it shields the genital branch of the GNF nerve from perineural scarring and direct contact with the mesh. In females, avoiding cutting the round ligament of the uterus is essential to reduce the risk of injury to the genital branch of the GNF nerve [23, 24]. Ensuring that the deep inguinal ring is not excessively narrow is crucial to avoid direct contact between the II nerve and the mesh. Fixing the mesh to the IOM aponeurosis prevents the involvement or entrapment of the muscular segment—the most vulnerable part—of the IHG nerve. Fixing the mesh to the periosteum of the pubic bone should be avoided because it can cause chronic pain. The suture should be placed at the inguinal ligament at the level of the deep inguinal ring to prevent femoral nerve entrapment [1, 6, 7, 12, 13, 20].

### Pragmatic neurectomy

#### Recommendation 3

Pragmatic neurectomy should be performed in cases of nerve injury, risk of entrapment, or nerve interference at the time of mesh fixation.

**Fig. 4:**
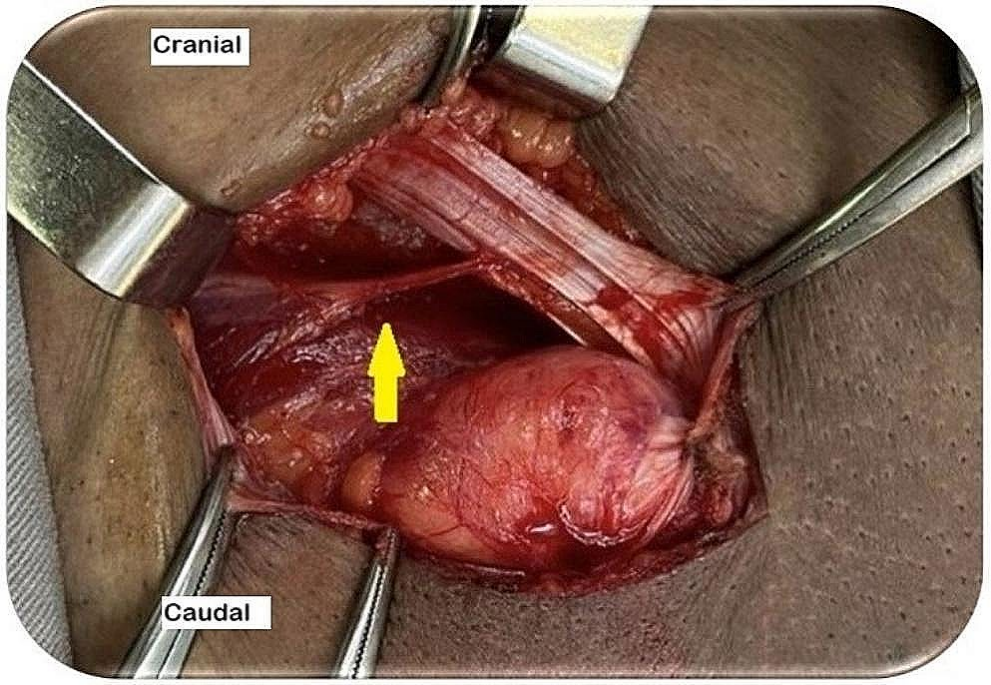
Right inguinal region post-EOM aponeurosis opening. Identification of the IHG nerve in topography that interferes with mesh placement (yellow arrow: IHG nerve). (source: author)

**Fig. 5:**
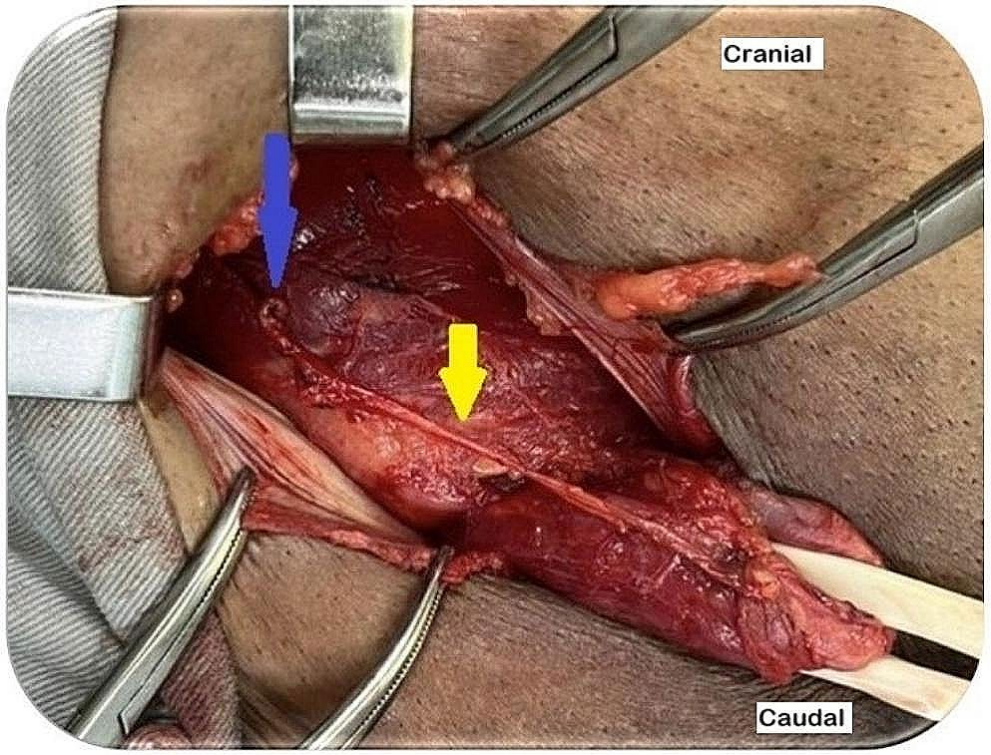
Right inguinal region post-EOM aponeurosis opening The IHG nerve after dissection in its extension with proximal stump ligation using absorbable sutures (pragmatic neurectomy). (source: author). The yellow arrow shows the dissected IHG nerve, and the blue arrow shows nerve stump ligation with absorbable sutures

Pragmatic neurectomy has been adopted with the aim of minimizing the risks of nerve injury [1, 6, 12, 23, 24]. (Fig. 4) The II and IHG nerves are the most commonly used in neurectomy. Nerve injuries can be complete or partial (axonotmesis, neurotmesis, or neuropraxia). Axonotmesis, neurotmesis, and complete nerve injury can cause neuromas and increase the risk of chronic pain. A pragmatic neurectomy should be performed to prevent neuromas [22–24]. The injured nerves must be resected along their entire length, as distally and proximally as possible, and ligated with absorbable sutures to prevent exposure of the myelin sheath (Fig. 5). The proximal nerve stump (II or IHG) is implanted within the IOM fibers, thus preventing adhesion of the nerve stump to the inguinal ligament or IOM aponeurosis. Adhesion of the neural stump to these structures can result in nerve traction when walking or moving the hip, potentially triggering postoperative neuralgia. The proximal stump of the genital branch of the GNF nerve is ligated with slight traction to remove the stump from the deep inguinal ring [24].

Prophylactic neurectomy of the II or IHG nerve is not recommended because it does not reduce the incidence of chronic pain but may increase the risk of sensory loss. However, if necessary, a prudent surgeon should discuss the risks and benefits of neurectomy with the patient [24–28].

### Spermatic cord structure management

#### Recommendation 4

Protecting the cremasteric fascia and visualizing the external spermatic vein (blue line).

The SC must be isolated from the inguinal floor without injury using Mixter or Kelly forceps. Digital dissection by elevating and circling the SC with a finger should be avoided because of its traumatic nature, which increases the risk of cremasteric fascial injuries. The SC should be mobilized en bloc with all its structures and released from the inguinal floor approximately 2 cm beyond the pubic tubercle. It should be mobilized with a Penrose drain, as materials such as gauze or pads increase local trauma and the risk of injury to the genital branch of the GNF nerve. The cremaster muscle fibers are transversely incised at the level of the deep inguinal ring to identify an indirect hernia or cord lipoma. If a lipoma is detected, it must be resected. The cremaster muscle should not be resected and exposed, as in the original technique, because it can injure small blood vessels and paravasal nerves, leading to torsion of the vas deferens and increasing the risk of ischemic orchitis, chronic pain, and burning sensation after ejaculation [1, 6, 8, 16, 20, 23]. When associated with pragmatic neurectomy, cremaster resection can be considered in cases of inguinal scrotal hernia with SC enlargement, cremaster hypertrophy, or a dilated inguinal ring. This surgical tactic aims to reconstruct the internal inguinal ring to avoid chronic pain; however, insufficient evidence exists according to the consensus on the management of inguinal-scrotal hernias [29].

### Femoral canal assessment

#### Recommendation 5

Femoral canal assessment is mandatory to prevent missed femoral hernias.

**Fig. 6:**
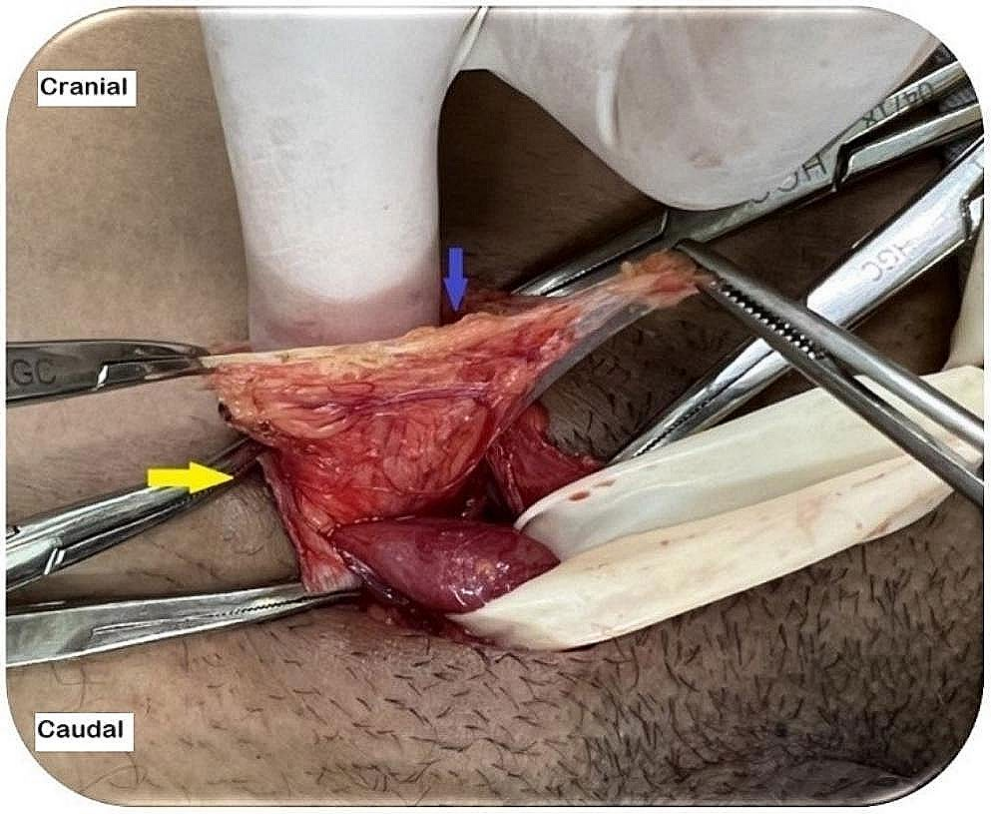
Femoral canal assessment by opening the indirect hernia sac (source: author). Yellow arrow: indirect hernia sac; blue arrow: femoral canal analysis through the hernia sac

The canal is assessed through the space of Bogros or the hernial sac. In indirect hernias, the hernial sac is opened, and the femoral region is analyzed from within the hernial sac (Fig. 6). In direct hernias, a small opening is made in the transversalis fascia to expose the femoral canal [6, 8, 16]. 

**Fig. 7:**
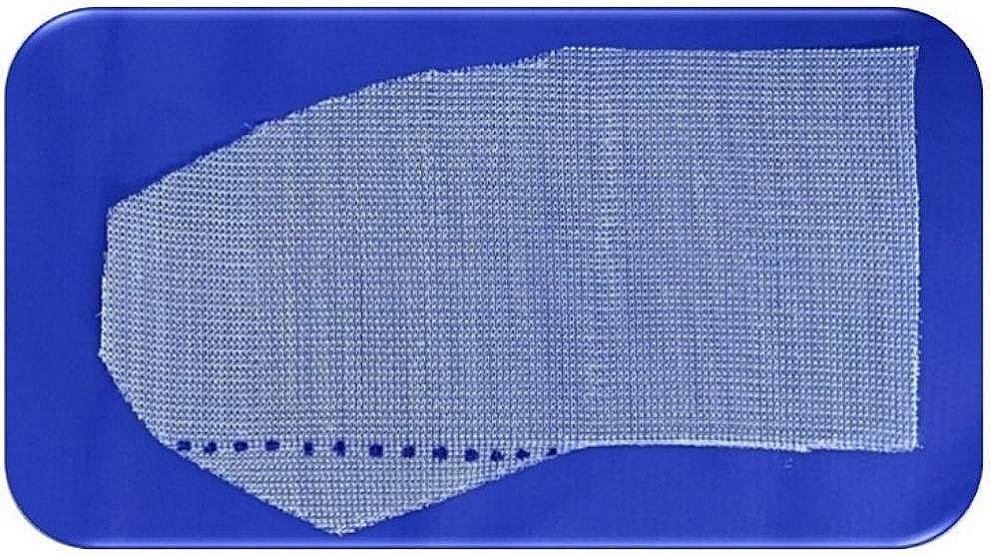
Mesh with a triangular extension used in cases of femoral hernia. (source: author). The triangular part is attached to Cooper’s ligament, and the dotted part to the inguinal ligament

If a femoral hernia is identified during these maneuvers, it must be corrected simultaneously by changing the mesh shape. The mesh must exhibit a triangular extension at its lower edge (Fig. 7). After opening the posterior wall and reducing the hernia content, this extension is sutured to Cooper’s ligament, and the body of the mesh is sutured to the inguinal ligament (dotted line) [6, 8, 16, 18].

### Hernial sac management

#### Recommendation 6

Management of the hernial sac depends on the type of hernia: indirect, direct, or inguinal-scrotal.

**Fig. 8:**
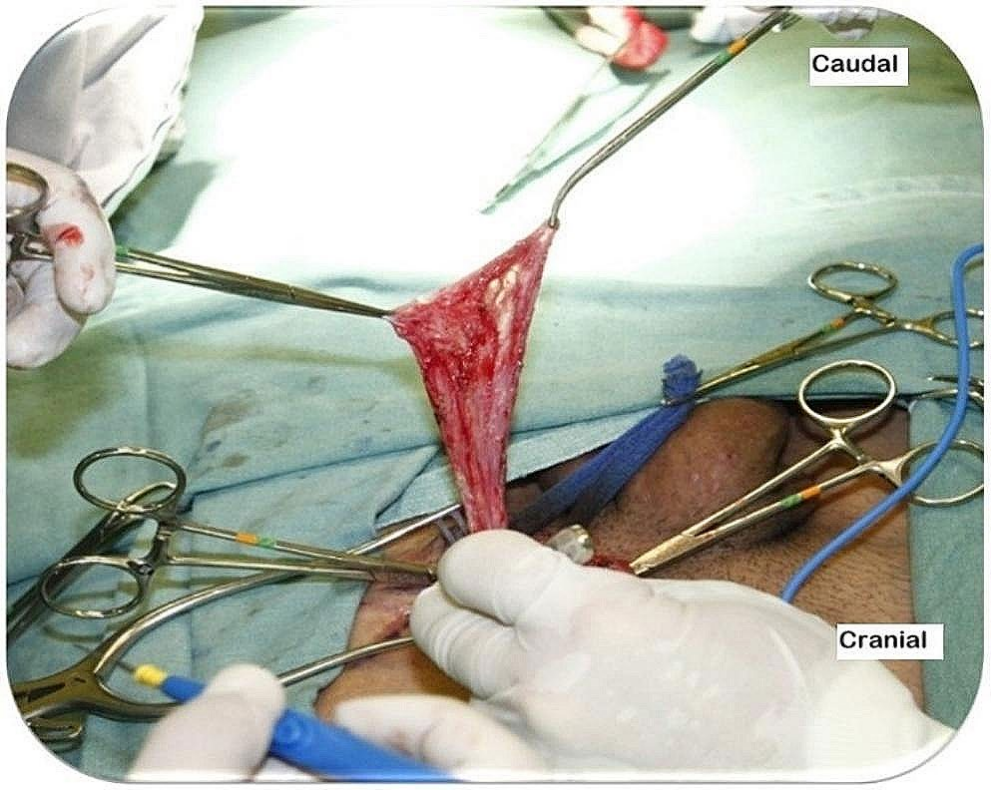
Left inguinal region. Indirect hernial sac dissected from the SC to be reduced to the abdominal cavity without ligation. (Source: Photograph courtesy of Dr. Sergio Roll)

In indirect hernias, the hernial sac is released from the SC beyond the neck and inverted or reduced into the abdominal cavity without ligation. This approach minimizes the risk of chronic pain without increasing recurrence or complication rates (Fig. 8). Ligation should be avoided, as it increases the likelihood of postoperative pain [3, 5–9, 29–32].

The transversalis fascia can be sutured in direct hernias using continuous or purse-string stitches with absorbable threads. This suture restores the anatomy and facilitates mesh placement [3, 5]. 

Inguinal-scrotal hernias are approached slightly differently. Ideally, the hernial sac should be reduced en bloc. When this is not feasible, the hernia should be transected at the midpoint of the inguinal canal, leaving the distal part of the sac open and opening the anterior wall to reduce the risk of hydrocele [29].

### Mesh

#### Recommendation 8

Choosing the mesh with appropriate characteristics and format.

**Fig. 9:**
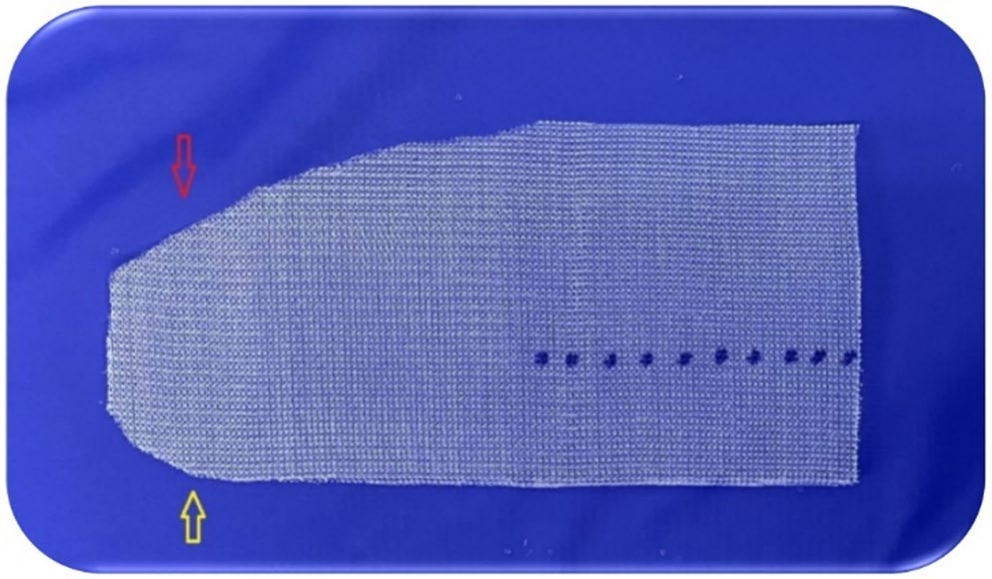
Mesh shape used in the Lichtenstein technique. (source: author)

The standard mesh used in Lichtenstein surgery resembles a footprint. The medial end of the mesh has a sharp curve (on the great toe side of the foot), which fits the angle between the inguinal ligament and anterior rectus sheath, and a wider curve that spreads over the rectus sheath (Fig. 9). This mesh format has remained the same since its introduction in 1993 and is used in more than 95% of cases, regardless of the hernia size [1, 12, 13, 16]. 

One side has a sharp curve to fit between the inguinal ligament and the rectus sheath (yellow arrow), whereas the other has a wider curve to fit over the rectus abdominis sheath (red arrow). The blue dotted line shows where to cut the two edges of the mesh (top 2/3 and bottom 1/3).

The use of a mesh in IH surgery is well-established and reduces the risk of recurrence. Nevertheless, the impact of prostheses on the etiology of pain and foreign body sensations cannot be ignored. Commercially available meshes are composed of different materials and exhibit several characteristics (e.g., pore size, weight, pore effectiveness, strength, and elasticity). In IH repair, weight and porosity are crucial. The mesh weight is directly contingent on the weight of the polymer, which is measured in grams/m^2^ (grammage). These meshes are categorized as ultralow, low, medium, and heavy grammages [33–39]. Light or low-grammage meshes weighing < 40 g/g/m^2^ tend to induce less inflammation and foreign body sensation. Porosity influences the biological behavior of the mesh, affecting the formation of fibrotic scar tissue and resistance to infection. Large-pore meshes are characterized by pores ranging from 1 to 1.5 mm [1, 12, 33–39]. 

The optimal recommendation for IH repair using the Lichtenstein technique is to employ monofilament synthetic meshes characterized by low weight, large pores, and a tensile strength exceeding 16 N/m^2^. Such meshes reduce the incidence of chronic pain and foreign-body sensation without increasing recurrence. Nevertheless, studies with extended follow-up periods have shown that the incidence of chronic pain appears to be equivalent between low- and heavy-weight meshes in later stages [1, 12].

The autoimmune syndrome induced by adjuvants is worth taking note of. This is a relatively unknown complication associated with the use of polypropylene mesh, particularly in women. Its diagnosis is complicated because it presents with non-specific symptoms such as fatigue, myalgia, arthralgia, and neurological disorders triggered by prosthesis use. The recommended treatment involves the removal of the prosthetic material [40].

### Fixation

#### Recommendation 8

Correct fixation prevents chronic pain, mesh mobilization, or bending and reduces the risk of recurrence.

**Fig. 10:**
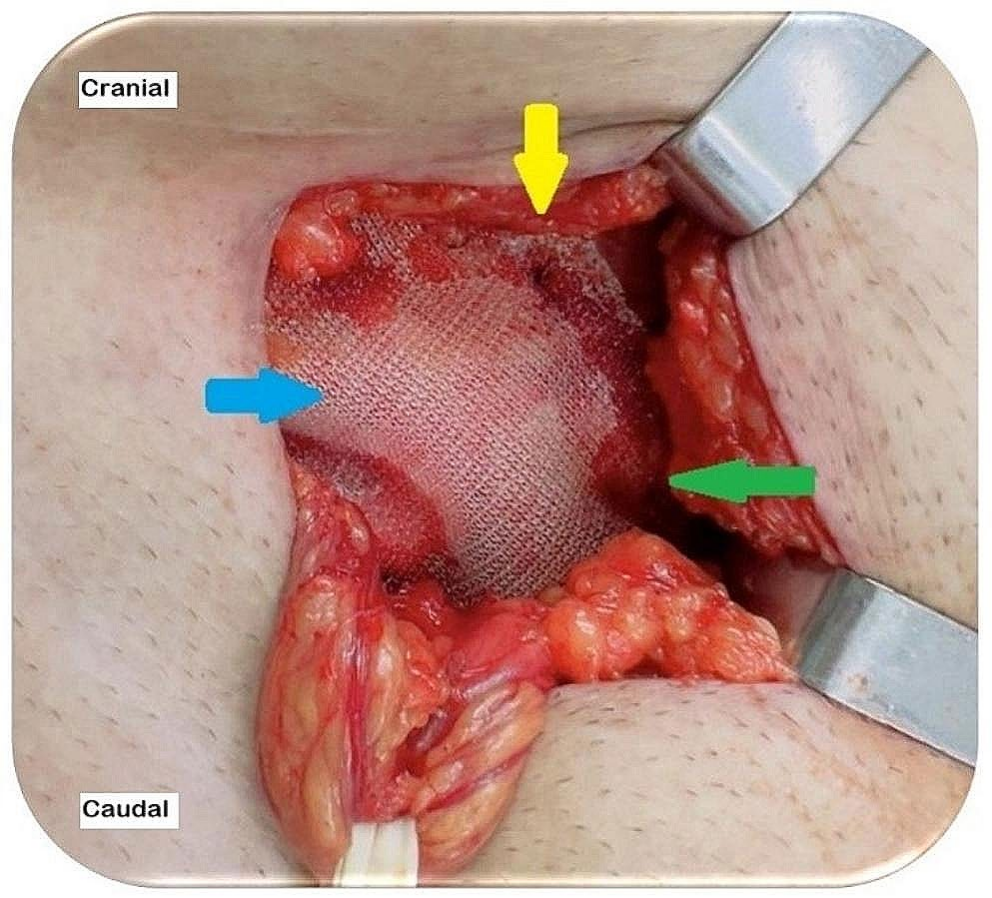
Right inguinal hernia. Fixing the mesh with loose absorbable stitches. (source: author). Yellow arrow: a stitch in the IOM aponeurosis; green arrow: a stitch in the rectus abdominis sheath; blue arrow: mesh slightly relaxed after fixation

**Fig. 11:**
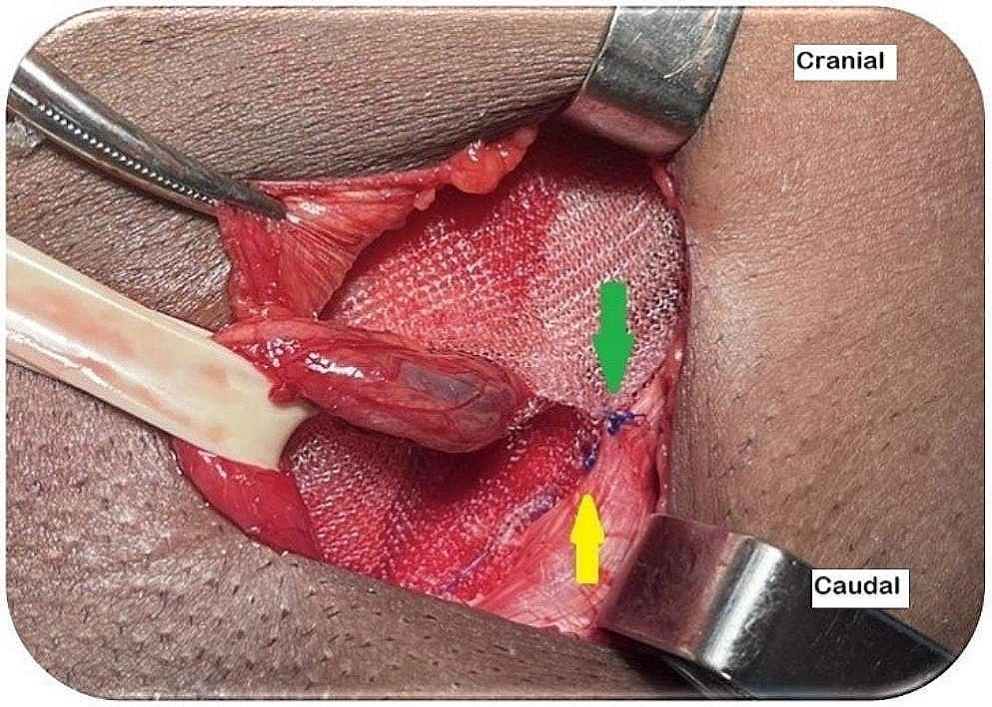
Left inguinal region. Mesh fixation to the inguinal ligament with monofilament nonabsorbable suture. (source: author). Yellow arrow: last stitch of the continuous suture at the level of the deep inguinal ring; green arrow: fixation of the lower edges of the mesh to the inguinal ligament juxtaposed to the last stitch of the continuous suture

Proper fixation of the mesh is crucial for the Lichtenstein technique. The medial portion of the mesh should be secured with two interrupted absorbable stitches: one in the rectus abdominis sheath and the other in the IOM aponeurosis at the level of the deep inguinal ring. The IHG muscular segments must not be included. These stitches should be loosely tied to avoid tissue necrosis and chronic pain (Fig. 10) [41]. The mesh must be affixed to the inguinal ligament with a continuous nonabsorbable monofilament suture, passing the needle three to four times (Fig. 11). Beginning at the reflex inguinal ligament and avoiding the periosteum of the pubic bone, the suture ends at the level of the deep inguinal ring. The lower mesh edges should overlap (i.e., the upper end must cover the bottom), be fixed with nonabsorbable sutures to the inguinal ligament, and be juxtaposed to the last knot of the continuous suture (Fig. 11) [1, 4, 6–8, 13, 14, 16, 41]. 

Anterior mesh repairs exhibit no significant differences regarding infection or recurrence between the fixation methods (traumatic or atraumatic). Atraumatic fixation, such as that using glue, sealants, cyanoacrylate, or self-fixing meshes appears to reduce postoperative pain only in the initial stages. Self-fixing meshes demonstrated only to lower operative time without clinical advantages over sutured mesh fixation [1, 20, 42]. Therefore, atraumatic fixation warrants only a weak recommendation according to the international consensus on hernia management [1].

### Recurrence prevention

#### Recommendation 9

Proper fixation, appropriate mesh size, overlapping, wide dissection, and anatomical knowledge are essential to reduce the risk of recurrence.

The primary recurrence sites are the pubic region (owing to the lack of adequate mesh overlap on the pubic tubercle) and the region close to the deep inguinal ring (owing to the lack of crossing of the mesh edges behind the SC) for direct and indirect hernias, respectively [4–8, 12–14, 16]. 

The mesh dimensions (7.5 × 15 cm) should adequately cover vulnerable areas. A 1 cm incision may be necessary to properly fit the mesh in the inguinal region in some patients. To comprehensively cover this area, the mesh overlaps should extend 2 cm beyond the pubic symphysis, 5–6 cm lateral to the internal inguinal ring, and 3–4 cm beyond the inguinal triangle [3, 4, 6, 8, 13, 14, 43]. The mesh should be slightly relaxed or dome-shaped to accommodate the transversalis fascia protrusion when the patients exert themselves, thus compensating for mesh countertraction (Fig. 10). Crossing the mesh edges behind the SC is crucial to prevent recurrence lateral to the internal inguinal ring. The mesh must be cut into the distal region to create two edges. The upper and lower edges should be 2/3 and 1/3 of the mesh width, respectively. The upper edge must cover the lower edge [4–8, 12, 16, 42, 43]. Furthermore, the lower edges must be sutured to the inguinal ligament using a nonabsorbable monofilament thread and juxtaposed to the last knot of the continuous suture (Fig. 11). This fixation technique cannot be altered in patients undergoing surgery using a flat mesh. However, this type of fixation can be skipped when pre-loosened meshes are used. In such cases, the two edges can be sutured together with 0.5–1 cm of crossing, thus simplifying the procedure [6].

### Surgical convalescence

#### Recommendation 10

Convalescence duration.

Patients should resume their activities without restriction as soon as they feel able or feel no pain during usual activities, typically three to five days after surgery [1, 8, 12]. In initial studies by Lichtenstein et al. (1989), patients were encouraged to cough intraoperatively and perform the Valsalva maneuver to assess the strength of the repair without compromising surgical outcomes related to recurrence [5, 8, 9]. Early return to activities does not adversely affect recurrence rates. Conversely, sedentary patients have a recurrence rate that is twice that of active patients [41].

## Discussion

Recent extensive academic debates have discussed the superiority of IH repair techniques (open vs. laparoscopic) [16]. The literature indicates highly comparable recurrence, chronic pain, and complication rates [16, 20, 44–48]. Therefore, defining a technique as the gold standard is challenging [20]. Comparing personal and institutional results of the Lichtenstein technique remains challenging owing to individual variations within the surgical method [5]. A recent study published in Brazil shows interesting results regarding individual differences in this technique and highlights the need for increased knowledge regarding many technical steps used in Lichtenstein surgery [15]. Therefore, systematizing the surgical approach may facilitate result evaluation and comparison with other techniques.

Studies of chronic pain have proliferated with advances in neuroanatomical insights into the inguinal region. Chronic pain can affect up to 69% of patients undergoing IH surgery [20, 21]. Recent findings indicate that 12–16% of patients have moderate to severe pain six months post-surgery [1, 12, 22]. Given that approximately 20 million patients undergo IH surgery annually globally, the prevalence of chronic pain is assumed to be significant. Thus, the outcomes of patients with chronic pain should be improved [1, 12].

Identifying inguinal nerves is a logical, pivotal step to mitigate chronic inguinal pain post-herniorrhaphy since this technical step may reduce the incidence of chronic pain to less than 1% [21]. This approach serves a dual purpose: it protects the nerves and allows for a neurectomy in case of nerve interference with mesh placement [23]. Despite a lack of comprehensive anatomical and surgical understanding of the inguinal region, proficiency in inguinal neuroanatomy and inguinal canal topography is essential for all IH surgeons [2]. Chronic pain prevention measures must be incorporated into the surgical procedure, including wide dissection, preservation of the neural covering fascia, atraumatic manipulation, nerve identification, and pragmatic neurectomy [6, 7, 13].

Acquiring knowledge of the technical principles of the Amid-modified Lichtenstein technique and surgical strategies to prevent chronic pain can improve surgical outcomes, significantly impacting public health and patients’ quality of life [16].

The ten recommendations: neuroanatomical assessment, chronic pain prevention, pragmatic neurectomy, protecting the cremasteric fascia and visualizing the external spermatic vein (blue line), femoral canal assessment, hernial sac management, choosing the mesh with appropriate characteristics and format, proper fixation, recurrence prevention and convalescence duration are practical ways to achieve a safe and successful procedure. We fell that following these recommendations can improve surgical outcomes using the Lichtenstein technique.

## Conclusion

Comprehensive knowledge of the technical steps of Lichtenstein technique is essential for IH surgery. Given the evolution and modifications of the technique since its initial description, this study lists ten recommendations as a practical guide for surgeons to enhance the outcomes of patients undergoing inguinal hernioplasty using the Lichtenstein technique.

## Data Availability

All data generated or analysed during this study are included in this published article.
